# Perspectives on the Genetic Associations of Ankylosing Spondylitis

**DOI:** 10.3389/fimmu.2021.603726

**Published:** 2021-03-05

**Authors:** B. Paul Wordsworth, Carla J. Cohen, Connor Davidson, Matteo Vecellio

**Affiliations:** ^1^ Nuffield Department of Orthopaedics, Rheumatology and Musculoskeletal Sciences, University of Oxford Institute of Musculoskeletal Sciences, Oxford, United Kingdom; ^2^ Botnar Research Centre, Nuffield Orthopaedic Centre, Oxford, United Kingdom; ^3^ Wellcome Centre for Human Genetics, University of Oxford, Oxford, United Kingdom

**Keywords:** epigenetics, aetiology, pathogenesis, spondyloarthropathy, interleukin-23

## Abstract

Ankylosing spondylitis (AS) is a common form of inflammatory spinal arthritis with a complex polygenic aetiology. Genome-wide association studies have identified more than 100 loci, including some involved in antigen presentation (*HLA-B27*, *ERAP1*, and *ERAP2*), some in Th17 responses (*IL6R, IL23R, TYK2*, and *STAT3*), and others in macrophages and T-cells (*IL7R, CSF2*, *RUNX3*, and *GPR65*). Such observations have already helped identify potential new therapies targeting IL-17 and GM-CSF. Most AS genetic associations are not in protein-coding sequences but lie in intergenic regions where their direct relationship to particular genes is difficult to assess. They most likely reflect functional polymorphisms concerned with cell type-specific regulation of gene expression. Clarifying the nature of these associations should help to understand the pathogenic pathways involved in AS better and suggest potential cellular and molecular targets for drug therapy. However, even identifying the precise mechanisms behind the extremely strong HLA-B27 association with AS has so far proved elusive. Polygenic risk scores (using all the known genetic associations with AS) can be effective for the diagnosis of AS, particularly where there is a relatively high pre-test probability of AS. Genetic prediction of disease outcomes and response to biologics is not currently practicable.

## Introduction

Ankylosing spondylitis (AS) is the archetype of a group of inflammatory disorders known as spondyloarthropathies (SpA) because they often affect the spine (axial skeleton). Other forms of SpA (e.g., psoriatic arthritis, reactive arthritis and the enteropathic arthropathies associated with inflammatory bowel disease—IBD) also often involve the axial skeleton (axSpA) but sometimes just affect the peripheral joints (peripheral SpA). Any part of the spine may be involved in AS but the SI joints are the most commonly affected sites early in the disease. The demonstration of radiographic sacroiliitis is a formal prerequisite for the diagnosis of AS but may take many years to be apparent on plain films. Therefore, to diagnose early AS or axSpA (considered together here although there are semantic differences), magnetic resonance imaging (MRI) is preferred since it can detect the early inflammatory phase of the disease potentially many years before radiographic changes become apparent on X-rays (1, 2). AS is one of the commonest forms of arthritis in the developed and developing world with a prevalence of up to one in 200 in Western Europe but it is much less common in some other parts of the globe, such as sub-Saharan Africa where its low prevalence generally reflects the low frequency of the immune response gene HLA-B27 with which it is so strongly associated—see below (3). In this review, we focus on AS and axSpA (defined by imaging criteria—either radiographs or MRI) as might be diagnosed using the algorithm presented by Taurog et al. (4). Unfortunately, despite increased awareness of the disease and the diagnostic utility of MRI, the diagnosis of AS is still missed all too often; only one-third of cases are diagnosed within a year of the onset of symptoms, and there is typically a delay of more than 6 years before the diagnosis is established (5, 6).

**Figure 1:**
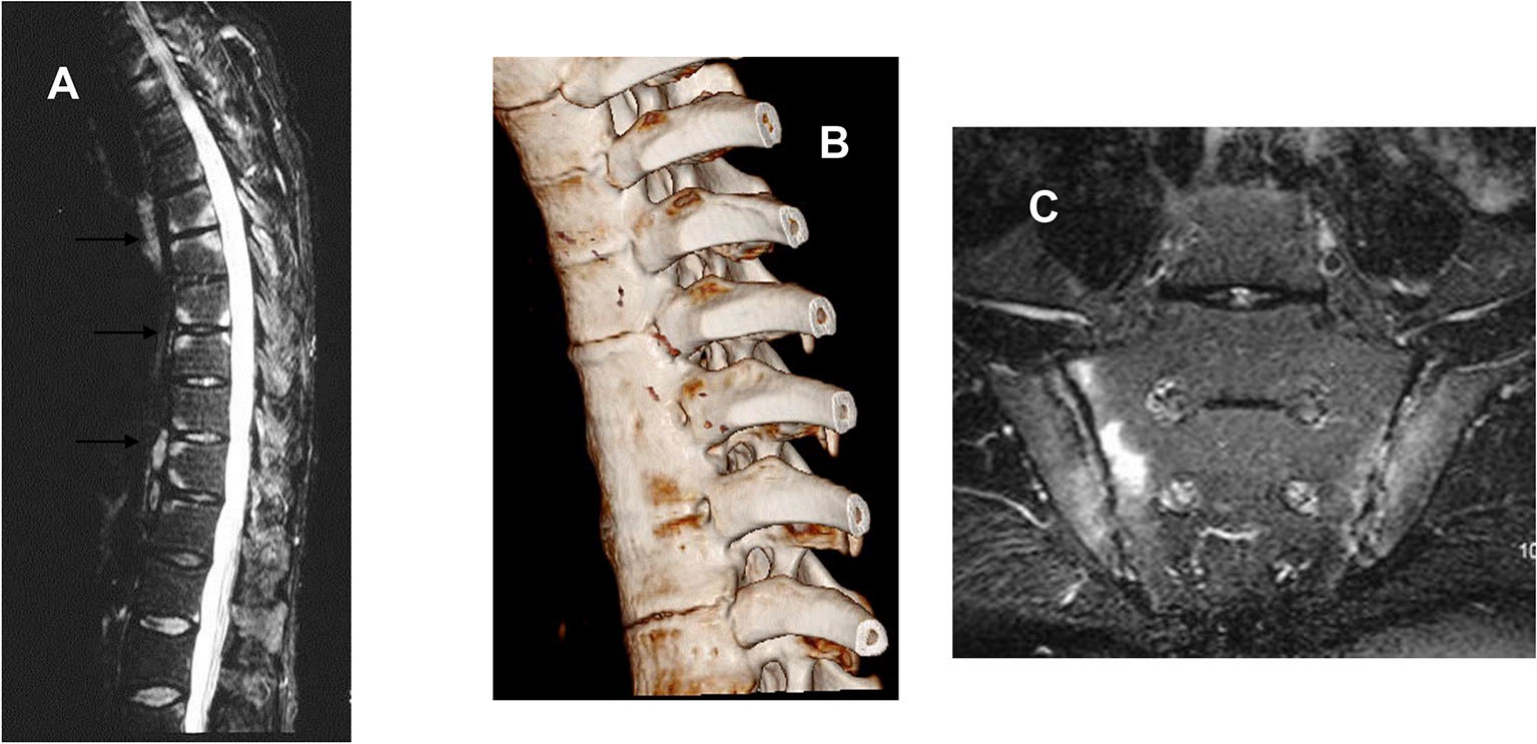
**(A)** Sagittal magnetic resonance image of the thoracic spine of a 44-year-old man with active ankylosing spondylitis, showing high signal on these T2-weighted images consistent with inflammation at the vertebral corners consistent with the attachment of vertebral ligaments and discs. **(B)** Computed tomographic reconstruction of the thoracic spine of a 25-year-old man with AS since the age of 12. There is clear bony fusion between the adjacent vertebrae and also at the costovertebral joints. **(C)** Bilateral sacroiliitis shown by MRI (STIR sequence) worse on the sacral side of the right SI joint.

In contrast to the inflammation of the joint lining (synovitis) associated with many other arthropathies, such as rheumatoid arthritis, the characteristic pathology of AS is enthesitis. The entheses are anatomical sites that have evolved to tolerate heavy mechanical loads, such as fibrocartilaginous joints (including the SI joints), the osseous insertions of ligaments and tendons, and joint capsules. In AS, inflammation at these sites initially causes bone erosion but this is often followed by new bone formation, which creates “syndesmophytes” that bridge between adjacent vertebrae in the spine causing bony fusion (ankylosis). Over time this can lead to complete loss of spinal movement and the classic “bamboo spine” appearance on radiographs characteristic of the most severe cases (**Figure 1**). Some years ago, Sherlock and his colleagues shed some light on why the entheses might bear the brunt of the pathological attack when they demonstrated the presence of CD3^+^ CD4^-^ CD8^-^ lymphocytes resident at the entheses expressing the interleukin (IL)-23 receptor (IL23R), and that a form of SpA resembling AS could be initiated in mice simply by liver-specific over-expression of IL23 alone without other cells being recruited to the affected tissues (7). Recently, γδ T-cells of both the Vδ1 and Vδ2 subsets have been demonstrated at the entheses that can be induced to produce IL-17, in the case of Vδ2 cells without the expression of IL23R (8). The relevance of IL23-driven pathways to the development of AS has also been amply demonstrated by numerous genetic associations with components of this pathway (see below).

**Figure 2:**
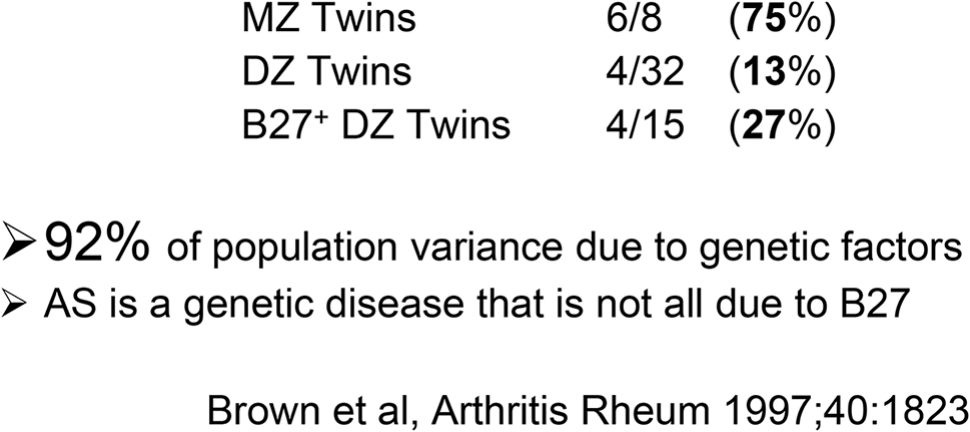
Studies of concordance for AS in UK twins recruited through the National Ankylosing Spondylitis Society. The clear difference on concordance rates between MZ twins and DZ twins is highly indicative of a major genetic component, which can only partly be explained by the influence of HLA-B27.

As with many other common diseases, the nature versus nurture debate regarding the aetiology of AS has long been a source of interest and speculation. Of course, increased familial recurrence can reflect either environmental or intrinsic factors but the absence of obvious temporal clustering of cases within families and the fact that the disease tends to start at a broadly similar age (typically between 20 and 40 years of age) is more suggestive of genetic than environmental influences. It was the particularly strong familial nature of the disease that prompted Derek Brewerton (at the suggestion of his rheumatology colleague Dudley Hart at the Westminster Hospital) to look for genetic risk factors in AS rather than rheumatoid arthritis in the 1970’s. By then it was already apparent that the pattern of AS recurrence risk among relatives of increasingly distant relatedness (very pronounced reduction in risk from first-degree to second-degree relatives, with more gradual reduction thereafter) was more consistent with a polygenic risk than either a monogenic or oligogenic contribution (9, 10). Despite this, such was the strength of the association between AS and the transplant antigen HLA-B27 (11, 12) that many erroneously assumed that AS was a monogenic disease. The classic way of investigating the genetic component of a disease by twin studies reveals a highly significant genetic contribution to AS, and one in which HLA-B27 is the major but by no means the only factor (**Figure 2**) (13). Armed with this limited but convincing information and the enthusiastic support of Sir John Bell and Mark Lathrop at the newly instituted Wellcome Trust Centre for Human Genetics a number of us from around the world therefore set out in the 1990’s to try to identify at least some of the other genes that were involved. In this brief review we discuss selected examples of the progress that has already been made towards this goal and how this has helped to pin down some of the pathological processes involved in AS. We discuss some of the innovative methods that have been used to identify new genetic associations with AS and the problems in interpreting these associations at a functional level. We include brief discussions of how these findings could inform future drug target discovery and play a role in the diagnosis of AS, and personalizing therapeutics for individual patients.

## Genome-Wide Association Studies in AS

Prior to the late 1990s efforts to identify any non-HLA genes contributing to AS were limited to studies of so-called “candidate” genes for which there was (usually but not invariably) a compelling biological reason for why they might be involved. Naturally enough (given the association with HLA-B27), many of these candidates were broadly “immunological” in nature and, equally unsurprisingly, they were generally unrewarding. The transition to genome-wide approaches was perhaps somewhat offensive to some scientists, because it was essentially not “hypothesis-driven” in the classic Popperian philosophical sense—other than that we proposed that there were genes out there to be discovered. The initial studies in AS, based on a form of genetic linkage analysis of affected relative pairs proved too blunt an instrument for the job (despite the huge amount of work involved in recruiting several hundred affected sibling pairs and their nuclear families). Beyond demonstrating linkage to gene(s) in the major histocompatibility complex on chromosome 6 not very much else came up and certainly nothing that was categorically associated with AS even after applying meta-analysis (14–16). Worse still, it was obvious that this type of analysis had very little power to refine chromosomal intervals to the level of identifying individual genes and/or the polymorphisms in them that were disease-causing variants. It was not until technical advances allowed the application of much larger numbers (~500,000) of genetic markers known as single nucleotide polymorphisms (SNP) spanning the entire genome that the field really started to move on. Nonetheless, there were some exciting surprises even before this grand-scale technological revolution was fully in place. The following are just a few examples from the first decade of GWAS in AS.

### Early Successes: Endoplasmic Reticulum Associated Aminopeptidase 1

The first GWAS in AS was published in 2007 as part of a broader attempt by the Wellcome Trust Case-Control Consortium to identify the genetic component of several common complex diseases, including cardiovascular disease, bipolar disease, inflammatory bowel disease, rheumatoid arthritis, tuberculosis, autoimmune thyroid disease, multiple sclerosis, and breast cancer (17). The number of AS cases was relatively small (~1,000) and the number of SNPs was modest (~14,500, of which 3,000 were in the major histocompatibility complex - MHC). Although the SNPs were gene-targeted non-synonymous variants (i.e., amino acid changing) this only gave a coverage of about one SNP per two gene loci, on average. By chance, one of the genes that registered association in this study had been allocated rather more than its fair share of SNPs—*ERAP1* (endoplasmic reticulum aminopeptidase 1 involved in processing peptide antigens for presentation by MHC class I molecules) had 5 non-synonymous (coding) SNPs. To this day *ERAP1* remains one of the most interesting and strongest associations (p~10^-50^) with AS outside the MHC (18–21). It is one of a family of aminopeptidases involved in the progressive cleavage of single amino acids from the amino-terminal end of peptides transported *via* the TAP (transporter associated with antigen processing) prior to associating with nascent MHC class I molecules. ERAP1 is crucial in shaping the available peptide repertoire, not only by providing peptides of the optimal length (8–9 amino acids) but also by influencing their amino-terminal residues that affect their binding to individual HLA allotypes, such as HLA-B27. A number of fascinating subsequent discoveries have been made about the nature of this genetic association with AS and the functions of ERAP1.

1. The association with *ERAP1* is synergistic with HLA-B27. Only around 84 per cent of AS cases in the UK are HLA-B27 positive and the association of AS with *ERAP1* is restricted to those who are HLA-B27 positive (20). Interestingly, HLA-B27 negative AS is associated with another aminopeptidase—*ERAP2—*which is adjacent to *ERAP1* on chromosome 5 but in a separate linkage disequilibrium block. Given the clear functional interdependence of MHC class I molecules and these aminopeptidases it is perhaps unsurprising that there should be such obvious genetic interaction but there are actually remarkably few similar examples to date in the literature. Indeed, it was this synergy between HLA-B27 and ERAP1 that prompted others to look successfully for similar MHC/ERAP interactions in psoriasis, a condition with well-described genetic overlap with AS and SpA (22). Subsequently, similar findings have also been described in Behcet’s syndrome between *ERAP1* and HLA-B*51 (23).
2. *ERAP2* actually turns out to be associated both with HLA-B27 positive and negative AS (although it needs rather highly powered studies to prove it). There is a high frequency *ERAP2* null allele that results in about a quarter of Europeans having no functional ERAP2 although precisely how this affects susceptibility to AS is not currently known (24).
3. Altering the expression of ERAP1 or ERAP2 has a profound impact on the repertoire of peptides bound to MHC class I molecules, including HLA-B27 (25, 26). But how this relates to the pathogenesis of AS is also unknown. Any potential “arthritogenic peptide” remains highly elusive.
4. *ERAP1* polymorphisms that afford protection against AS are common loss-of-function variants with reduced aminopeptidase activity that are also likely to influence this repertoire (18, 27, 28). Consequently, there would appear to be scope for developing small molecule inhibitors of ERAP1 (and possibly other aminopeptidases) in the quest for new therapies for the prevention or treatment of AS.

### A Credible GWAS Hit: Interleukin-23 Receptor

The same early GWAS (17) that identified *ERAP1* also revealed the first evidence of association between AS and the *IL23R* locus on chromosome 1, encoding the IL23-specific component of the of the heterodimeric IL23 receptor (the other component, IL12RB1, can also combine with IL12RB2 to form the IL12 receptor) (29). In the main part of this study, the initial strength of the association was weak (as is often the case in such relatively poorly powered studies), but it was subsequently amply confirmed and strengthened (20, 21, 30). Further, this *IL23R* association is recapitulated in other diseases, such as psoriasis and inflammatory bowel disease (IBD), which commonly occur in individuals with AS and/or their relatives, highlighting a degree of shared genetic background between these conditions (22, 31). The main SNP primarily associated with AS, psoriasis and IBD (*rs11209026*) causes a loss-of function mutation in the cytoplasmic tail of IL23R that reduces IL-17 and IL22 production by Th17 effector cells (32, 33) and modulates responses to pattern recognition receptors (34). These findings suggest that IL-23 driven pathways are implicated in AS, a finding supported by the subsequent identification of several other genetic associations with components of the Th17 lymphocyte developmental pathway, including *IL6R*, *TYK2, STAT3, IL1R1/2*, and *IL12B* (encoding the p40 fragment of IL12 that dimerises either with p35 in IL12 or p19 to form IL23). Coffre et al. suggest that the effector functions of Th1 and Th17 cells are affected by multiple variants at genetic loci associated with the IL23-driven pathway, including *IL23R, IL12B, CCR6, IL17A/F, IFNG, IL12RB2, TBX21*, and *RORC* (35).

These findings support the case for targeting various components of the IL23 pathway as a means of treating AS. Further, since many of the same genetic associations are also found in psoriasis and IBD (36) similar therapeutic strategies might also be expected to be fruitful in these conditions. However, the results have proved somewhat unpredictable and indicate substantial complexity in the relevant biological pathways and their involvement not only in their effects in the various related forms of SpA but also on the associated skin and bowel disease. Thus, targeting of IL-17 (the main pro-inflammatory cytokine associated with terminally differentiated Th17 cells) with the therapeutic monoclonal antibodies secukinumab or ixekizumab has proved highly successful in AS (37, 38) and psoriasis (39) but not IBD (40). Targeting the p40 subunit common to both the IL23 and IL12 receptors (thereby blocking both IL12 and IL23) has proved disappointing in AS and axial SpA (41, 42) in contrast to its efficacy in psoriasis and IBD (43, 44). Finally, despite its success in treating psoriasis, psoriatic arthritis and IBD (45, 46) the therapeutic antibody risankizumab, which targets the p19 fragment of IL23, is ineffective in AS (47). It is therefore interesting that AS does not show the same genetic association with IL23 as psoriasis (36), perhaps suggesting that Il-23 itself is important in psoriasis while IL23R and downstream signalling pathways are rather more relevant to the pathogenesis of AS.

### A Second Association at the IL23R Locus?

More detailed genomic studies have revealed other associations near *IL23R* independent of *rs11209026* in the intergenic region between *IL23R* and the neighbouring *IL12RB2* gene (tantalisingly encoding the IL12-specific component of the IL12 receptor - see above). The associated SNP - rs11209032 - lies in a regulatory region, including a transcription factor binding-site for TWIST1, and appears to increase Th1 cell differentiation but, so far, its role in the pathogenesis of AS is unclear (48, 49). The International Genetics of AS (IGAS) Immunochip study in 2013, which fine mapped ~200 loci of known importance in immune responses and inflammation, revealed that such complex associations with more than one SNP independently associated with AS at a given locus are not uncommon (21).

### Other “Hits” With Immunological Relevance: IL7R (IL7 Receptor α Chain) CSF2 (Granulocyte-Macrophage Colony-Stimulating Factor), and GPR65 (G-Protein Coupled Receptor 65)

Unsurprisingly the IGAS Immunochip study identified or confirmed genome-wide associations with many other loci implicated in immune/inflammatory conditions (because, after all, that was what the “Immunochip” was designed to do). For example, the “suggestive” AS association with *rs6897932* in *IL7R* mirrored similar genome-wide significant associations of *IL7R* with multiple sclerosis and primary biliary sclerosis (21, 50, 51). The “C allele” affects differential splicing of the 6^th^ exon in the transmembrane domain of IL7R and increases the amount of both membrane-bound and soluble IL7R. Soluble IL7R increases the half-life of IL7, which plays a key role in T-cell immunity. Synovial fluid monocytes from patients with SpA have increased levels of IL7R and a transcriptome profile that overlaps with IL-7-induced gene sets (52). Type 3 innate immune cells expressing IL7R are also increased in the synovial tissues of patients with SpA, and these cells produce GM-CSF (granulocyte-macrophage colony-stimulating factor) after *in vitro* stimulation (53). Targeting GM-CSF with therapeutic antibodies has already been shown to be effective in rheumatoid arthritis (54) and would therefore appear to be an obvious target in SpA as well (one such antibody—namilumab—is currently under investigation in the Namaste Trial—ClinicalTrials.gov Identifier NCT036226658).

Further evidence supporting a role for GM-CSF in AS also comes from the genetic association with *GPR65* (encoding a G-protein-coupled receptor involved in proton sensing) in the IGAS Immunochip study (21). Although it was not appreciated at the time GPR65 plays an integral role in regulating GM-CSF expression. Single cell genomics reveals that it is also crucial to the pathogenicity of Th17-cells in murine experimental allergic encephalomyelitis (55). Th17-cells are pleiotropic; there are increased numbers of GM-CSF secreting CD4^+^ and CD8^+^ lymphocytes in the synovium and peripheral blood of patients with SpA, and also increased numbers of IL-17A^+^/GM-CSF^+^ double-positive CD4^+^, CD8^+^, γδ and NK cells. GM-CSF^+^CD4^+^ lymphocytes express GPR65 irrespective of whether they co-express IL-17A (53). Silencing *GPR65* in primary CD4-cells results in reduced GM-CSF expression and so it may also be an important potential therapeutic target for SpA.

### A Plausible Association Without Functional Corroboration: NOS2 (Inducible Nitric Oxide Synthase)

The Immunochip study showed a convincing peak of association with SNPs upstream of the *NOS2* gene (21), which has previously been associated with susceptibility to infectious diseases, such as leishmaniasis, and inflammatory diseases in mice (56). *NOS2* is also associated with IBD where its expression in the gut mucosa is highly dysregulated (57). In contrast to mice, human macrophages appear not to have the same inducible up-regulation of *NOS2* (despite the application of many different conditions and stimuli, in the hands of one of us—CD). The *NOS2* genetic association appears solid and lies in a region upstream of the gene likely to have regulatory functions BUT (1) “Is this region actually regulating *NOS2* or another gene?”, (2) “Are the conditions necessary to induce *NOS2* in human macrophages highly specific and different from those that we have tried so far?”, or (3) “Is the effect on *NOS2* expression manifest in a different cell type from those we have explored to date?”. With regard to the latter, it is interesting that around two-thirds of patients with AS have subclinical inflammation of the terminal ileum so perhaps the gut mucosa might be a more productive place to look (58).

### A Strongly Associated Locus With Relationship to Immune Cell Development: RUNX3 (Runt-Related Transcription Factor 3)

The challenge of identifying a mechanistic explanation for genetic disease associations is hard enough when there is a clear functional effect arising from a protein-coding change, as in the case of *rs30187* in *ERAP1* or *rs11209026* in *IL23R*, or for that matter HLA-B27. Far more often the lead SNP in such associations lies outside the coding sequence, most likely in regions concerned with the regulation of gene expression—but “Which genes?” and “How are they regulated?” are huge issues. Such *cis*-regulatory elements are most likely to control the activity of neighbouring or nearby genes, but their influence could extend even megabases down the chromosome. These issues are well exemplified by the *RUNX3* association with AS.

RUNX3 is one of the family of multifunctional RUNX transcription factors that play key roles in the development and differentiation of many cell types, including many immune phenotypes. It has been strongly associated with AS by GWAS (20), and the lead SNP mapped more accurately in the Immunochip study to a region with characteristics of an enhancer upstream of the promoter (21, 59). Careful examination of this region reveals that there are at least two independent neighbouring AS-associated SNPs that affect the binding of different transcription factors. Further, despite the fact that they are only 500 base pairs apart, these two distinct SNPs appear to exert their influence in different cell types—*rs4648889* in CD8+ T-cells and *rs4265380* in monocytes (60). The challenge now is to translate this into a better understanding of the regulatory framework of genes involved and how this affects the pathogenesis of AS. Fortunately the science of “genomics” now provides a wealth of publicly available data relating to the regulation and expression of genes in specific cell types that facilitate these investigations. These include (1) eQTL (expression quantitative trait loci) mapping that relates gene expression to particular SNPs in particular cell types, such as monocytes (61), (2) areas of “open” chromatin (DNAse 1 hypersensitivity sites), (3) transcription factor binding-sites and (4) other chromatin modifications, such as histone methylation or acetylation, that indicate the activity status of genes and their enhancers (62, 63). All of these can potentially be used to cross-reference functional gene activity at the cellular level with disease-associated SNPs to pursue the ultimate aim of discovering relevant disease pathways and how they might be therapeutically manipulated.

In our lab, we have so far demonstrated that the *RUNX3* AS-associated SNP rs4648889 (above) mediates differential allelic binding of two regulatory factors/complexes to a putative enhancer in the region upstream of the promoter: (1) the transcription factor interferon regulatory factor (IRF) 5, which binds preferentially to the AS-protective “G” allele; and (2) components of the nucleosome remodeling and deacetylase (NuRD) complex (one of the four major ATP-dependent chromatin remodeling complexes that function as transcriptional repressors) bind preferentially instead to the AS-risk “A” allele at rs4648889 (64). Further work is necessary to confirm the functional consequences of this SNP on gene expression and the network of genes involved but preliminary experiments suggest that IRF5 knockdown in CD8+ T-cells reduces the expression of interferon gamma. Discovering new drug targets by this type of reverse genetics represents a daunting challenge that will require many different approaches and techniques. Identification of the disease-associated SNPs by statistical techniques is hard enough but further progress towards a mechanistic explanation for these GWAS associations will undoubtedly require: (1) precise identification of the primary functional genetic variants involved (within an AS-associated LD block); (2) their effects on gene expression in specific cell types (transcriptomics); (3) their effects on protein translation (proteomics); and (4) how these vary in response to different stimuli (metabolomics). The majority of AS-associated loci exert only very small effects on predisposition to the disease, most likely through quite subtle regulatory effects on gene transcription. These will inevitably still need to be assessed in more complex cellular systems and relevant animal models. Nonetheless, even at this early stage of the investigation of *RUNX3* there are already hints that both CD8+ T-cells and monocytes might constitute plausible cellular targets for intervention in AS (60). Credible molecular targets have yet to emerge.

### A Replicated GWAS Hit Without an Obvious Explanation: ANTXR2 (Anthrax Toxin Receptor 2)

Among the numerous genetic associations with AS are many that defy obvious explanation. The SNPs lying in an extended linkage disequilibrium block including the entire *ANTXR2* gene is an excellent example. The initial positive association found by the Triple A (Australo-Anglo-American) spondyloarthritis consortium (TASC) has been amply replicated in independent studies but it has been difficult to decide precisely which SNP is most closely associated with the disease (19, 65). Our limited knowledge of the biology of the protein does little to offer an explanation for its genetic association with AS. In addition to its role as a potential receptor for the anthrax toxin it appears to be involved in capillary morphogenesis. *ANTXR2* mutations also cause the rare monogenic hyaline fibromatosis syndrome (On-line Mendelian Inheritance in Man catalogue number—228600), in which there are widespread subcutaneous nodules and other internal organ involvement, but none of this gives many clues as to whether or how it might be involved in AS. So far it is not even clear whether these SNPs are actually involved in the actions of *ANTXR2* or another gene in the vicinity. This is a common issue in providing mechanistic explanations for many GWAS “hits”.

### AS Genetics in Clinical Practice

#### Diagnostic Testing

A role for HLA-B27 in the diagnosis of AS is well established but its use should be implemented with care; the sensitivity and specificity of HLA-B27 testing is clearly related to the pre-test probability that an individual might have AS. Used as a screening test for AS on all individuals with low back pain in the community it is quite unhelpful, but if limited to individuals with clinical features suggestive of the condition it is very useful. People in whom the condition is suspected can be placed in a “suspicious” group according to their responses to a few simple questions. These include: (1) Chronicity (low back pain > 3 months), (2) Alternating buttock pain (indicative of SI joint inflammation), (3) Improvement with gentle exercise or anti-inflammatory analgesics, (4) Back pain interfering with sleep in the second half of the night, (5) Onset aged less than 40 years of age, (6) Affected first-degree relative, (7) Presence of co-morbidities known to be associated with AS, such as psoriasis, IBD or uveitis. Individuals with positive responses to these questions have a much higher pre-test probability of AS than others with low back pain in the community, and in those with 4 or more positive responses an additional positive HLA-B27 result may increase the likelihood of AS to over 90%. This can be further increased by the finding of SI joint inflammation on MRI. However, even with the combination of clinical questions, HLA-B27 testing and MRI the diagnosis is either missed or incorrect in about 5% of cases (6). The diagnosis is accurately made in only a third of patients in the first year of symptoms and is frequently delayed by 6 years or more (5). Brown et al. (66) have nicely reviewed the state of the art relating to biomarker development in AS, including genetic testing. They highlight the utility of HLA-B27 testing but suggest that polygenic risk scores (PRS), which additionally use all the other SNPs associated with AS, can give an even better positive predictive value (67). Using this approach, they and others have convincingly demonstrated that using 110 SNPs with reported genome-wide association to AS (including HLA-B27) is significantly more discriminatory than HLA-B27 alone in the diagnosis of AS. However, the difference is relatively small and of unproven clinical value (68). In contrast, a few well-chosen questions (see above) designed to identify those with high likelihood of AS/axSpA prior to implementing any sort of genetic testing are worth their weight in gold.

#### Prognosis

Prediction of the prognosis and outcomes of treatment in AS are long-term goals that could be facilitated by genetics since we already know that the severity of the disease is highly heritable and certainly not determined exclusively by HLA-B27 status (69). There is some evidence that outcomes from biologic therapies are better in HLA-B27 positive patients and that positive responses to secukinumab may be influenced by the *ERAP1* risk allele at *rs30187* (37, 66). However, these conclusions have been drawn from small studies and clearly require replication. We have also investigated a SNP in *TNFRSF1A* (encoding the p55 TNF Type 1 receptor) for its potential to influence not only susceptibility to AS but also its severity and responsiveness to anti-TNF biologics. The “G” allele of rs1800693 is associated with susceptibility to multiple sclerosis but protection against AS (20, 70, 71). It causes skipping of exon 6 resulting in a truncated soluble form of the protein with potential anti-inflammatory properties, mimicking the action of the anti-TNF fusion protein etanercept; this is particularly interesting given the possible association between anti-TNF biologic therapy and central nervous system demyelination (70, 72). However, the *rs1800693* polymorphism in *TNFRSF1A* neither appears to affect the severity of AS nor its response to anti-TNF biologics (73). In order to characterize such genetic influences on responses to therapy it may well be necessary to examine far larger case series than has been done to date.

### Longstanding Conundrums

#### Why Does Not Everyone With HLA-B27 Get AS?

The aetiology of AS clearly involves other genetic and/or environmental factors than just HLA-B27. Twin studies indicate its polygenic nature, which is one explanation for why only around 5% of those with HLA-B27 develop AS. Estimates of broad-sense heritability suggest that over 90% of the population variance can be attributed to genetic factors (13) but this does not preclude the involvement of common environmental influences, such as infections, in its aetiology. It merely suggests that any such extrinsic factors are likely to be so common (like certain viral infections)? that they do not greatly influence the population variance (at least in developed Western societies). Whether this is always the case is a moot point. There are some exceptions to the general rule that the prevalence of AS mirrors that of HLA-B27 in the population. Thus, in The Gambia in tropical west Africa AS is exceptionally rare (as it is in much of sub-Saharan Africa) (3), but in contrast to many other African countries the frequency of HLA-B27 in The Gambia is at least 6% (not so very different from ~8% in the UK). The low Gambian prevalence of AS was initially attributed to the existence of an unusual HLA-B27 variant—HLA-B*2703—with potentially different functional characteristics from the HLA-B*2705 allele, which is predominant in European populations (74). However, on closer inspection at least half of the B27-positive individuals in The Gambia actually carry the “European” HLA-B*2705 allele, making it far from rare in that population (75). Another explanation for the rarity of the condition in this population is therefore necessary: perhaps there is some other genetic factor in this population or, more likely, something different about the Gambian environment that affords protection against the disease.

#### What About the Gut?

There has been much interest in the possibility of a link between the gut and AS for many years. One of us remembers the excitement at The Middlesex Hospital in London after early reports that faecal carriage of *Klebsiella* sp. was associated with active disease. However, these studies provoked strong views on either side, particularly relating to whether this could be explained on the basis of cross-reactive “autoimmune” responses (76, 77). Nevertheless, many lines of evidence point towards gut involvement in SpA and much current research. For example, IBD is often complicated by various forms of peripheral and axial arthritis, the onset of which may be before, concurrent or afterwards. Curiously, there are quite distinct clinical features to these various forms of arthritis. The type 1 peripheral arthropathy of IBD (similar to reactive arthritis in its asymmetric, pauciarticular, predominantly lower limb distribution) is strongly associated with HLA-B27 as is the axSpA associated with IBD, but the former runs a course mirroring activity of the IBD in contrast to the axSpA, which is independent of IBD activity (78, 79). In the type 2 peripheral arthropathy of IBD (polyarticular, upper and lower limb distribution), joint disease activity is also not linked to activity of the IBD and it has a distinct immunogenetic profile (not associated with HLA-B27 but rather with HLA-B44 (80). In our sample of ~8,500 cases of AS from the UK there is co-existent clinically overt IBD in ~10–15%, which is at least partly due to their shared genetic background (36). In other studies, two-thirds of those with AS without overt IBD exhibit subclinical histological gut inflammation (81). There is also some circumstantial evidence from long-term observational studies that a minority of individuals with reactive arthritis (usually a self-limiting condition triggered by infection in the gut or urogenital tract) may progress over time to axSpA/AS (82). Attempts to identify specific causative agents in the gut, such as *Klebsiella* sp., have largely proved unsuccessful but there is still much interest in the potential role that the gut microbiome might play in AS and its potential role in mediating local and systemic inflammation in SpA [reviewed in (66, 83)]. Wholesale sequencing of gut bacteria suggests that the gut microbiome in AS can be distinguished from the normal population and may have some correlation with disease activity (84–88). However, whether these results are truly disease specific must also take into account that the HLA alleles associated with AS (and also those associated with rheumatoid arthritis) have a significant impact on the host gut microbiome in healthy individuals too (89).

#### What Is the Evidence for a Specific Antigenic Stimulus in AS?

The strong HLA-B27 association with AS suggests that adaptive immune responses are important in its pathogenesis but any “arthritogenic peptide(s)” has so far proved elusive. Evidence for antigen-driven specific immune responses in the HLA-B27 associated arthropathies, is not new (90, 91) but the development of high throughput sequencing to assess the T-cell receptor repertoire has seen a recent resurgence of interest. Of particular interest, TCR binding motifs from some patients with AS show similarities with those identified previously in individuals with reactive arthritis (92–95). There is also evidence of a significant increase in CD8+ T-cell clonotypes specific for the Epstein-Barr and cytomegalovirus (92). In this regard it is therefore interesting that recent studies have identified conventional CD4+ and CD8+ T-cells resident at the entheses in humans that have regulatory phenotypes and reactivity against common viruses, including cytomegalovirus (particularly CD8+ T-cells) (96).

#### What Is the Role of HLA-B27 in AS?

Fifty years after it was first described the mechanism(s) underlying the strong association of AS with HLA-B27 still requires a truly convincing explanation. We have certainly learned a lot about this molecule in the intervening years—crystal structure, the peptide repertoire it binds, its unusual folding characteristics, and interactions with receptors on innate immune cells—but where has this left us? Although there is some evidence of specific antigen presentation (see above) this is certainly inconclusive. Other theories have drawn on some of the atypical features of HLA-B27 among MHC class I molecules – in particular, its relatively slow folding and tendency to form homodimers. For a more detailed description of these theories the reader is referred elsewhere (97–99). Briefly, in addition to its role in antigen presentation HLA-B27 is unusual in its folding kinetics; unusual forms can accumulate in the endoplasmic reticulum causing an unfolded protein stress response, which can lead to IL23 production in dendritic cells. Similar responses have been observed in macrophages in the transgenic rat model of SpA (100). This theory provides a neat explanation for the apparent lack of antigen specificity in animal models of SpA (99) but is far from settled given the lack of evidence of UPR in gut epithelial cells from individuals with AS (100). HLA-B27 is also unusual in its ability to form homodimers or free heavy chains that can be recognized by killer-immunoglobulin-like receptors (KIR), which are mainly expressed on NK cells but also on CD4+ T-cells (101, 102). People with AS have a higher frequency of T-cells expressing this receptor and these are also polarized towards the Th17 phenotype that is associated with AS (97). Of interest, ERAP1 variants associated with protection against AS reduce HLA-B27 free heavy chain expression on monocytes and potentially reduce Th17 activity (103).

#### Bone Modeling and HLA-B27

Only a few tentative genetic associations that have been reported between AS and genes involved in bone modeling to date. Weak associations have been described with *RANK* (receptor activator of NF kappa B involved in osteoclast development) in Caucasians and *RANKL* (RANK ligand) in Chinese (104, 105). However, another recent paper suggests that HLA-B27 is involved in the activation of *TNAP* (encoding the enzyme alkaline phosphatase) in mesenchymal stem cells obtained from syndesmophytes of patients with AS. This led *in vitro* to accelerated mineralisation in a manner that was independent of the key osteoblast transcription factor RUNX2. Further, in an animal model, this process could be inhibited by bisphosphonates, a group of drugs commonly used in the treatment of osteoporosis, thereby holding considerable promise of a treatment that could retard the abnormal ossification and ankylosis associated with AS (106).

## Concluding Remarks

**Figure 3:**
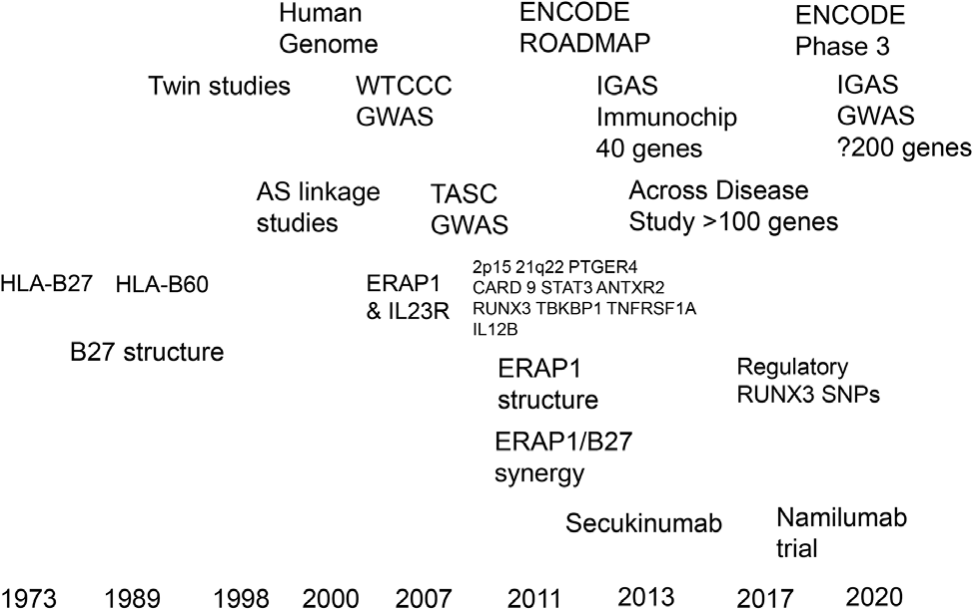
Timelines of progress in translating the genetics of ankylosing spondylitis towards therapeutics.

It may be argued that so far, we have actually learned more about the treatment of common diseases from studying rare, phenotypically severe, monogenic conditions than from the genetics of common polygenic diseases like AS. There have certainly been some spectacular successes. First, the development of therapeutic RANKL (receptor activator of NFκB-ligand) antibodies (denosumab) for the treatment of osteoporosis, for which the insights came from very rare osteolytic bone diseases (familial expansile osteolysis—OMIM 174810) affecting the RANK/RANKL axis of osteoclast development (107). Second, anti-sclerostin antibodies (romosozumab) have also been successfully developed for the treatment of osteoporosis (108), based on the observation that loss-of-function mutations in sclerostin (a bone morphogenetic protein antagonist) were responsible for massive accumulation of bone in the rare recessive disorder, sclerosteosis (OMIM 269500). It is unsurprising that polygenic diseases have proved harder nuts to crack. Nevertheless, much progress has been made in AS already thanks to a hugely collaborative global effort (**Figure 3**).

If we have learnt anything about the study of complex diseases in the past 20 years, it is that size matters when it comes to genetic studies. With the assistance of various international consortia, we can generate sample sizes that now have the power reliably to detect loci increasing the risk of AS by 5% or less. Similar efforts will probably be essential to identify any genetic influences on therapeutic outcomes. Novel strategies for identifying susceptibility genes include increasing the power of such studies by combining cohorts of genetically related diseases, such as AS, psoriasis, IBD and sclerosing cholangitis. Individual loci identified in this way can then be individually tested in the specific disease subsets. The number of loci incriminated in AS has been increased to more than 100 in this way (36). Efforts to increase the number of cases for these studies have continued, and it is hoped that the latest GWAS from the IGAS consortium will present data from ~ 20,000 cases in the next 12 months. Translating these results into therapeutic targets will remain problematic but continuing advances in the field of functional genomics hold much promise for progress in this field (109). Detailed analysis and discussion of these issues is beyond the scope of this review, so the interested reader is referred to the 30^th^ July issue of Nature that contains no fewer than 10 relevant articles on the subject [Nature 2020; vol 583: issue 7818]. As an example of what can be achieved, many of the associated genetic loci in another complex rheumatic disease—rheumatoid arthritis—have recently been shown to have complex chromatin interactions and effects on gene expression, specifically in T-cells. Further, using a multiomic approach, new genes not previously implicated by GWAS, such as *MYC* and *FOXO1* have been identified in the pathogenesis of the disease (110). In AS, even the original MHC association with HLA-B27 has been shown to be far more complex; there are numerous associations with both Class I and II alleles, and additional epistasis with ERAP1 (111). With a few exceptions (105–107, 112) most translational work in AS genetics has concentrated to date on its immunological and inflammatory contributions but, given that much of the pathology and the ensuing disability is caused by abnormal bone deposition, there is a strong case for investigating this aspect of the disease more intensively.

## Author Contributions

BPW and MV conceived the manuscript. BPW, MV, CD, and CJC drafted the manuscript. All authors contributed to the article and approved the submitted version.

## Funding

MV was funded by Versus Arthritis Career Development Fellowship 21428. CD was funded by Versus Arthritis DPhil studentship 22198. CJC was funded by Versus Arthritis project grant 20402. BPW is supported by the National Institute for Health Research Oxford comprehensive Biomedical Research Centre (A93081).

## Conflict of Interest

The authors declare that the research was conducted in the absence of any commercial or financial relationships that could be construed as a potential conflict of interest.
